# Case of Spina Bifida

**Published:** 1860-11

**Authors:** John Low


THE CHICAGO

MEDICAL JOURNAL.

VOL. III. November, 1860. NO. 11.

### Original Communications.

### ARTICLE 1.

### CASE OF SPINA BIFIDA—DEATH—POST MORTEM EXAMINATION.

This child, a female, was born February 8, 1860. Was well formed except a tumor in the lower part of the back. The tumor was situated directly over the spinal column, nearly circular, and a little larger in circumference than a silver half dollar; was soft, flat, and fluctuating, and but slightly elevated above the surrounding integument; the covering, membrane-like, translucent, a portion of it quite thin and transparent. On the covering there was a spot about the size and shape of a half almond, quite red and flesh-like. Thirty hours after, this spot was thinly covered with healthy-looking pus. The contents of the tumor appeared to be from one to two drachms of clear, serum-like fluid.

February 9.—Dressed the suppurating part with simple cerate.

Feb. 10.—Commenced compressing the tumor, by using a mixture of equal parts of collodion and castor oil, continuing the above-mentioned dressing to the suppurating spot. Functions of bowels and bladder well performed. The left lower extremity more relaxed than the right; the left foot scarcely if at all, sensitive to tickling.

Feb. 13.—No perceptible change except a slight increase in contents of tumor. Applied collodion undiluted.

March 1.—Suppurating spot healed; collodion continued. Child growing finely.

June 16.—Was called in to-day. (My note book goes on to say.) For the past few days the child has been peevish and fretful. On the surface of the tumor the suppurating spot has reappeared; it is quite opaque, with red and somewhat raised edges. This morning a considerable quantity (from two to three teaspoonfuls, the mother thought) of clear, water-like fluid gradually escaped from the inflamed surface. Now the child is not as fretful but does not like to be moved.

June 18.—Yesterday morning no oozing from the tumor. This morning fluid trickles away with tolerable freedom, cloths on the back of the child wet. Child quiet; takes the breast readily; bowels slightly constipated from the use of anodyne medicines; tumor dressed with simple cerate; collodion discontinued.

June 22.—Child died to-day at noon. Since last date it has grown constantly more fretful and restless; was somewhat quieted by Godfrey's cordial. About twenty-four hours before death began to have light convulsions. Slept the greater part of last night and had no convulsions. Upon awaking this morning the nervous disturbance manifested itself in almost constant jerkings of the lower extremities, the left more than the right. If the child fell asleep for a few seconds the jerking would cease, to be commenced again immediately she awoke.

The left lower extremity has had, except during the first week or two, a flexed position in all its joints, toes on the foot, foot on the leg, &c.; has been less sensitive to the touch than the right, but for the last few days both have been equally sensitive. The tumor is now sunken and apparently empty.

*Dissection.*—An incision was made through the tumor, extending several inches above and below it, along the spine. A few drops of pus were seen directly below the suppurating spot, and after cutting through a considerable depth of jagged soft tissue—apparently adipose and cellular—the spinal cord was seen lying in full view. The verteoral laminæ and processes were spread wide apart so as to form almost a flat surface with the posterior portion of the bodies of the vertebræ and the last two of the dorsal.

The spinal nerves appeared to pass off regularly through the intervertebral notches; the cord was somewhat lacerated, probably done in the process of dissection.

I presume the treatment by injection of iodine would not have answered in this case. In the beginning I gave an unfavorable prognosis, without, however, knowing the difference between those cases that admit of injection with hope of success and those that do not.

There was something noticeable, I think in the shape of the child's head; as she grew older it lost its round form, became flattened in front and laterally and correspondingly elongated in the direction of its vertex, but whether the spinal affection had anything to do with it I do not know. Albeit the child was as bright and intelligent as any child of the same age.

